# Serum branched-chain amino acids are associated with leukocyte telomere length and frailty based on residents from Guangxi longevity county

**DOI:** 10.1038/s41598-020-67010-9

**Published:** 2020-06-24

**Authors:** Ying Zhang, Qi Zhou, Ruiyue Yang, Caiyou Hu, Zezhi Huang, Chenguang Zheng, Qinghua Liang, Ranhui Gong, Xiaoquan Zhu, Huan Gong, Huiping Yuan, Chen Chen, Xianghui Li, Nan Zhang, Ze Yang, Liang Sun

**Affiliations:** 1 Peking University Fifth School of Clinical Medicine, Beijing, 100730 China; 2 The MOH Key Laboratory of Geriatrics, Beijing Hospital, National Center of Gerontology, Beijing, 100730 China; 3 Department of Neurology, JiangBin Hospital, Nanning, Guangxi 530021 China; 4 Office of Longevity Cultural, People’s Government of Yongfu County, Guilin, Guangxi 541899 China; 5 Department of Cardiothoracic Surgery, Guangxi Maternal and Child Health Hospital, Nanning, Guangxi 530005 China

**Keywords:** Geriatrics, Nutrition, Genetics, Cardiology

## Abstract

Branched-chain amino acids (BCAAs) and telomere length are biologically associated with healthy aging. However, the association between them and their interaction on frailty remain unclear in humans. Here, a cross-sectional study based on residents from Guangxi longevity county was conducted to investigate the association of serum BCAAs, peripheral leukocyte telomere length (LTL) and frailty. A total of 1,034 subjects aged 20 to 110 years were recruited in the study. The real-time qPCR method and a targeted metabolomics approach based on isotope dilution liquid chromatography tandem mass spectrometry (LC/MS/MS) method were used for measurement of LTL and BCAAs, respectively. A frailty score defined as the proportion of accumulated deficits based on 24 aging-related items was used assess the health status of elderly subjects. First, we found that a higher concentration of BCAAs was significantly associated with longer LTL only in middle-aged subjects, independent of age and BMI (*P* < 0.05). In the oldest-old subjects, we identified a significantly inverse association between BCAAs and frailty score (*P* < 0.001), even after adjustment for age and BMI (*P* < 0.05). Additionally, we recognized a statistically significant synergetic interaction between BCAAs and LTL on frailty score in the oldest-old subjects by the general linear model (*P* = 0.042), although we did not find any significant association between LTL and frailty score. In summary, our findings suggest a potentially protective effect of circulating BCAAs on LTL and frailty based on the subjects from longevity county in East Asia and indicate a potential synergetic interaction between BCAAs and LTL in healthy aging.

## Introduction

As a major phenotype of aging^1^, frailty is characterized by increasing vulnerability to external factors, a decreasing the ability to resist stress, and progressive loss of muscle performance^2,3^. Further, frailty has been reported to be a dominant risk factor for major cardiovascular endpoints and mortality in elderly^2^. Because frailty seems to be a dynamic process and also potentially reversible^3,4^, early recognition and intervention of frailty is important to achieve the goal of healthy aging. At present, the assessment of frailty is based on two methods: phenotypic method and cumulative health deficits method^1^. Compared to the phenotype approach, the accumulation of deficits approach is a more sensitive predictor for adverse health outcomes due to its finer graded scale and its multidimensionality^5^ and more suitable to provide a mechanistic understanding of the aging phenomenon^6^.

Branched-chain amino acids (BCAAs) are essential amino acids for the human, namely, valine (Val), leucine (Leu) and isoleucine (Ile). In addition to being proteogenic amino acids, BCAAs are also signaling molecules^7^. It was reported that BCAAs catabolism is a conserved regulator of physiological aging^8^. The mechanistic target of rapamycin (mTOR) signaling pathway is the most important one that BCAAs participate in. Sestrin2, a p53 target gene^9^, was reported to be a BCAAs sensor for the mTORC1 pathway^7^. Increased circulating levels of BCAAs have previously been reported to be associated with type 2 diabetes, insulin resistance, metabolic syndrome and cardiovascular risk in older people^10–13^,which conflicts with its potential anti-aging effects found in model organisms^14,15^. Recently, dietary restriction of BCAAs, with all other amino acids proportionately increased to maintain an iso-nitrogenous and iso-caloric diet, was reported to extend lifespan in *Drosophila*^16^. In addition, it was reported that serum BCAAs were significantly lower in frail participants compared to non-frail participants^2^.

Peripheral leucocyte telomere length (LTL) is recognized as a biomarker of cellular aging and biological aging and is influenced by many factors (e.g., genetic, epigenetic, life style and environment). Telomere shortening activates p53-mediated cellular growth arrest, senescence and apoptosis and induces metabolic and mitochondrial compromise^17^. It has been reported to be associated not only with chronological age but also age-associated pathologies. Recently, it has been proposed that frailty may lead to premature apoptosis in cells with normal length telomeres^18^. There are also many studies exploring the correlation between telomere length (TL) and frailty in the elderly, but the conclusions are inconsistent, and especially the data for the oldest-old are lacking^19^.

Yongfu County, locating in Guangxi Provience, has been qualified as a longevity town by the Geriatric Society of China in 2007^20^. Based on the local residents, we have conducted a longitudinal follow-up study for more than 10 years focusing on factors affecting longevity and healthy aging. Although BCAAs and TL are biologically associated with healthy aging, the association between them and their interaction on frailty remain unclear in elderly, especially in the long-lived individuals who are an excellent model for healthy human aging^21^. In light of this, a cross-sectional study based on residents from Guangxi longevity county was conducted to investigate the association between serum BCAAs, LTL and frailty.

## Results

Of the 1,034 included subjects, 478 (46.2%) were men, and 627 (60.6%) are the oldest-old aged from 86 to 110 years old. The mean (standard deviation, SD) concentrations of BCAAs, Val, Leu and Ile were 484.4 (103.8), 232.5 (46.1), 176.0 (45.3) and 75.9 (18.7) μmol/L, respectively. It is observed that the basic characteristics of the oldest-old subjects and middle-aged subjects are significantly different (Table 1). Finally, the calculation of frailty score was based on the 597 oldest-old subjects, due to some missing values. Generally, frailty score in the highest quartile of BCAAs is significantly lower than that of the lowest quartile (Fig. 2; Table S1).

**Table 1:** Characteristics of study participants^a^.

	Overall	the Oldest-old subjects	Middle-aged subjects
Number	1034	627	407
Age, years	73.1 ± 24.4	91.8 ± 4.1	44.1 ± 10.7
Male, n (%)	478 (46.2)	219 (34.9)	259 (63.6)
Uneducated, n (%)	465 (45.0)	439 (70.0)	26 (6.4)
Smoker, n (%)	402 (38.9)	105 (16.7)	297 (73.0)
Drinker, n (%)	439 (42.4)	130 (20.7)	309 (75.9)
BMI, kg/m^2^	20.2 ± 3.8	18.4 ± 3.2	22.9 ± 3.1
Overweight^b^, n (%)	138 (13.3)	24 (3.8)	114 (28.0)
Obesity^b^, n (%)	35 (3.4)	9 (1.4)	26 (6.4)
SBP, mmHg	150.2 ± 32.9	168.5 ± 27.1	122.1 ± 17.7
DBP, mmHg	79.0 ± 13.1	81.6 ± 13.4	74.9 ± 11.5
LTL	0.86 ± 0.54	0.81 ± 0.53	0.93 ± 0.53
BCAAs, μmol/L	484.4 ± 103.8	456.5 ± 90.0	526.7 ± 109.6
Val, μmol/L	232.5 ± 46.1	221.2 ± 41.0	249.5 ± 48.1
Leu, μmol/L	176.0 ± 45.3	162.9 ± 38.4	196.0 ± 48.0
Ile, μmol/L	75.9 ± 18.7	72.4 ± 17.3	81.2 ± 19.6

BMI, body mass index; SBP, Systolic blood pressure; DBP, Diastolic blood pressure; BCAAs, branched-chain amino acids; Val, valine; Leu, leucine; Ile, isoleucine; LTL, leukocyte telomere length.

^a^Data are mean ± SD for continuous variables and n (%) for categorical variables.

^b^Overweight was defined as 24 kg/m^2^ ≤ BMI < 28 kg/m^2^. Obesity was defined as 28 kg/m^2^ ≤ BMI.

**Figure 1:**
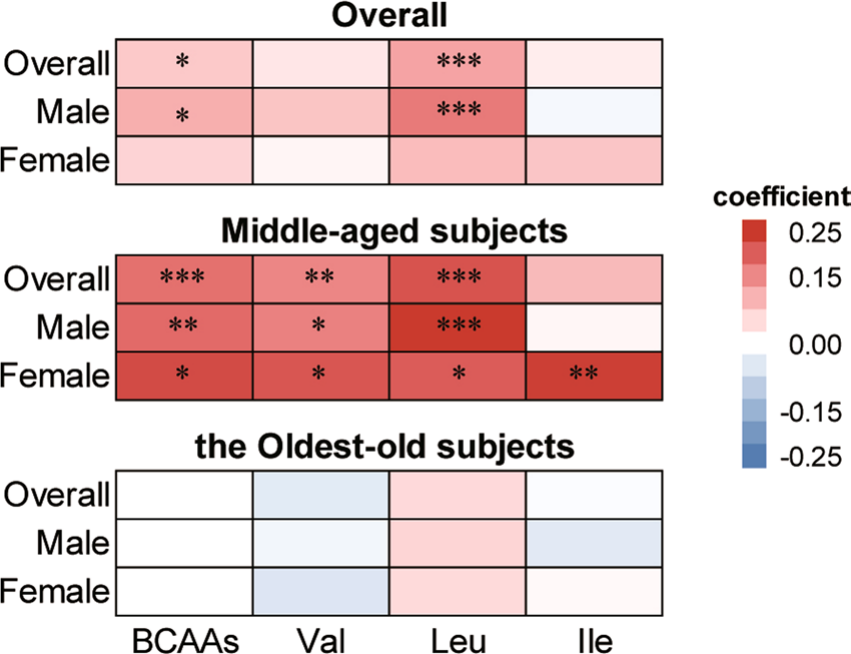
Heatmap of partial correlation coefficient between serum BCAAs and LTL stratified by gender. The color of red and blue represent positive and negative correlation respectively. The deeper the color, the stronger the correlation. The partial correlation coefficient was adjusted for age and BMI. BCAAs, branched-chain amino acids; Val, valine; Leu, leucine; Ile, isoleucine; BMI, body mass index. *P < 0.05; **P < 0.01; ***P < 0.001.

### Association between serum BCAAs and LTL

The partial correlation analysis showed a significantly positive association between BCAAs and LTL in all subjects (r = 0.1, *P* < 0.05). Further stratified analysis indicated that higher concentration of BCAAs, Val and Leu were significantly associated with longer LTL in both genders of middle-aged subjects independent of age and BMI, while the association between Ile and LTL was only found in female middle-aged subjects (Fig. 1; Table S2). However, no such significant association was found in the oldest-old subjects. No significant difference of serum BCAAs and LTL was observed between smoker and non-smoker, drinker and non-drinker in either the middle-aged subjects or the oldest-old subjects.

**Figure 2:**
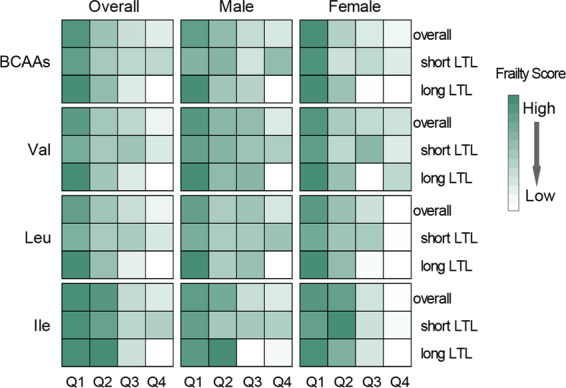
Heatmap of mean frailty score in quartiles of serum BCAAs stratified by gender and LTL in the oldest-old subjects. The darker the color, the higher the frailty score and the worse the health status. BCAAs, branched-chain amino acids; Val, valine; Leu, leucine; Ile, isoleucine; LTL, leukocyte telomere length.

### Association of serum BCAAs and LTL with frailty score

The significantly positive association between frailty score and age in all the oldest-old subjects (r = 0.3, *P* < 0.001) and both genders (*P* < 0.001) indicated that the score proved to be an effective parameter for evaluating aging-related health status.

A significantly inverse association between BCAAs and frailty score was found in all the oldest-old subjects (r = −0.20, *P* < 0.001) and both genders (*P* < 0.001). Each one SD increasement in BCAAs, Val, Leu, and Ile were remarkably associated with a reduction of mean frailty score in all the oldest-old subjects (Table 2) and both genders (Table S3). Further stratified analysis was performed due to the association between BCAAs and LTL, indicating that the inverse association of BCAAs with frailty was still established in subjects with long LTL. However, there was no significant association between LTL and frailty score (*P* = 0.362).

**Table 2:** The association between serum BCAAs and frailty score under different models stratified by LTL in the oldest-old subjects.

	LTL	Model 1			Model 2		
		β*	95%*CI*	*P*	β*	95%*CI*	*P*
BCAAs	overall	−0.04	−0.05, −0.02	<0.001	−0.03	−0.04, −0.01	<0.001
	short	−0.02	−0.04, −0.01	0.074	−0.02	−0.04, −0.01	0.174
	long	−0.05	−0.08, −0.03	<0.001	−0.04	−0.06, −0.02	<0.001
Val	overall	−0.04	−0.05, −0.02	<0.001	−0.02	−0.04, −0.01	0.002
	short	−0.01	−0.03, 0.01	0.283	−0.01	−0.03, 0.01	0.407
	long	−0.05	−0.07, −0.03	<0.001	−0.04	−0.06, −0.02	0.001
Leu	overall	−0.04	−0.06, −0.03	<0.001	−0.03	−0.04, −0.01	<0.001
	short	−0.03	−0.05, −0.01	0.031	−0.02	−0.04, −0.01	0.118
	long	−0.05	−0.07, −0.03	0.000	−0.04	−0.06, −0.02	0.001
Ile	overall	−0.03	−0.05, −0.02	<0.001	−0.03	−0.04, −0.01	<0.001
	short	−0.02	−0.04, −0.01	0.057	−0.02	−0.04, 0.01	0.139
	long	−0.04	−0.06, −0.02	<0.001	−0.03	−0.05, −0.02	0.002

BCAAs, branched-chain amino acids; Val, valine; Leu, leucine; Ile, isoleucine; LTL, leukocyte telomere length; 95% CI: 95% confidence interval; BMI, body mass index. *β refers to the average change of frailty score by one standard deviation increasement in amino acids concentration.

Model 1: crude. Model 2: adjusted for age and BMI.

### Interaction between serum BCAA and LTL for frailty score

A significant reduction in mean frailty score was found in the group with high serum BCAAs concentration and long LTL compared with the reference group with low serum BCAAs concentration and short LTL (*P* < 0.01). A statistically significant synergetic interaction between BCAAs, as well as Leu, and LTL on frailty score was recognized in the oldest-old subjects (*P* < 0.05) **(**Table 3**)**.

**Table 3:** Combined associations of serum BCAAs and LTL with frailty score in the oldest-old subjects.

	LTL	β^#^	95%*CI*	*P*	*P*_*interaction*_
**BCAAs**					
low	short	reference			
	long	0.02	−0.02, 0.06	0.263	
high	short	−0.04	−0.07, 0.00	0.059	0.042
	long	−0.07	−0.11, −0.03	<0.001	
**Val**					
low	short	reference			
	long	0.02	−0.02, 0.05	0.368	
high	short	−0.02	−0.06, 0.01	0.202	0.087
	long	−0.05	−0.09, −0.02	0.006	
**Leu**					
low	short	reference			
	long	0.02	−0.01, 0.06	0.218	
high	short	−0.03	−0.07, 0.01	0.154	0.038
	long	−0.06	−0.10, −0.02	0.002	
**Ile**					
low	short	reference			
	long	0.01	−0.03, 0.05	0.599	
high	short	−0.05	−0.09, 0.01	0.008	0.149
	long	−0.08	−0.12, −0.04	<0.001	

BCAA, branched-chain amino acids; Val, valine; Leu, leucine; Ile, isoleucine; LTL, leukocyte telomere length; 95%*CI*: 95% confidence interval. #β represents the difference in frailty score between each subgroup and the reference group. *P*_*interaction*_ was estimated from the general linear model by converting BCAAs and LTL into binary variables with a median split method.

## Discussion

To our knowledge, this is the first population-based study showing that elevated serum BCAAs concentrations are associated with longer LTL in middle-aged subjects and suggesting a potential synergetic interaction between serum BCAAs and LTL for the frailty of the oldest-old subjects.

It is widely accepted that the frailty of elderly subjects is closely related to aging-related skeletal muscle loss^22^. In addition, defects in mTOR signaling have been found in sarcopenic muscle^23^. It is also well-known that BCAAs are essential in the maintenance of muscle content and anabolic effects because they improve protein synthesis, and decrease the rate of protein degradation^24^. In our study, the significant negative association between BCAAs and frailty score was consistent with the other study^2^. However, the synergetic interaction between BCAAs and LTL for the frailty of the oldest-old has not been reported in other studies.

The reasons and mechanisms underlying this finding need to be further explored. In addition to being proteogenic amino acids, BCAAs are also signaling molecules^7^. It was reported that BCAAs catabolism is a conserved regulator of physiological aging^8^. The mTOR signaling pathway is the most important one that BCAAs participate in. Evidence from human embryonic kidney–293 T(HEK-293T) cells indicate that sestrin2 is a BCAAs sensor for the mTORC1 pathway^7^. And previous studies have demonstrated that the products of two p53 target genes, Sestrin1 and Sestrin2, connect genotoxic Stress and mTOR Signaling^9^. Moreover, the evidence also indicated that telomere shortening activates p53-mediated cellular growth arrest, senescence and apoptosis and induces metabolic and mitochondrial compromise^17^. These observations may help us understand the above findings.

**Figure 3:**
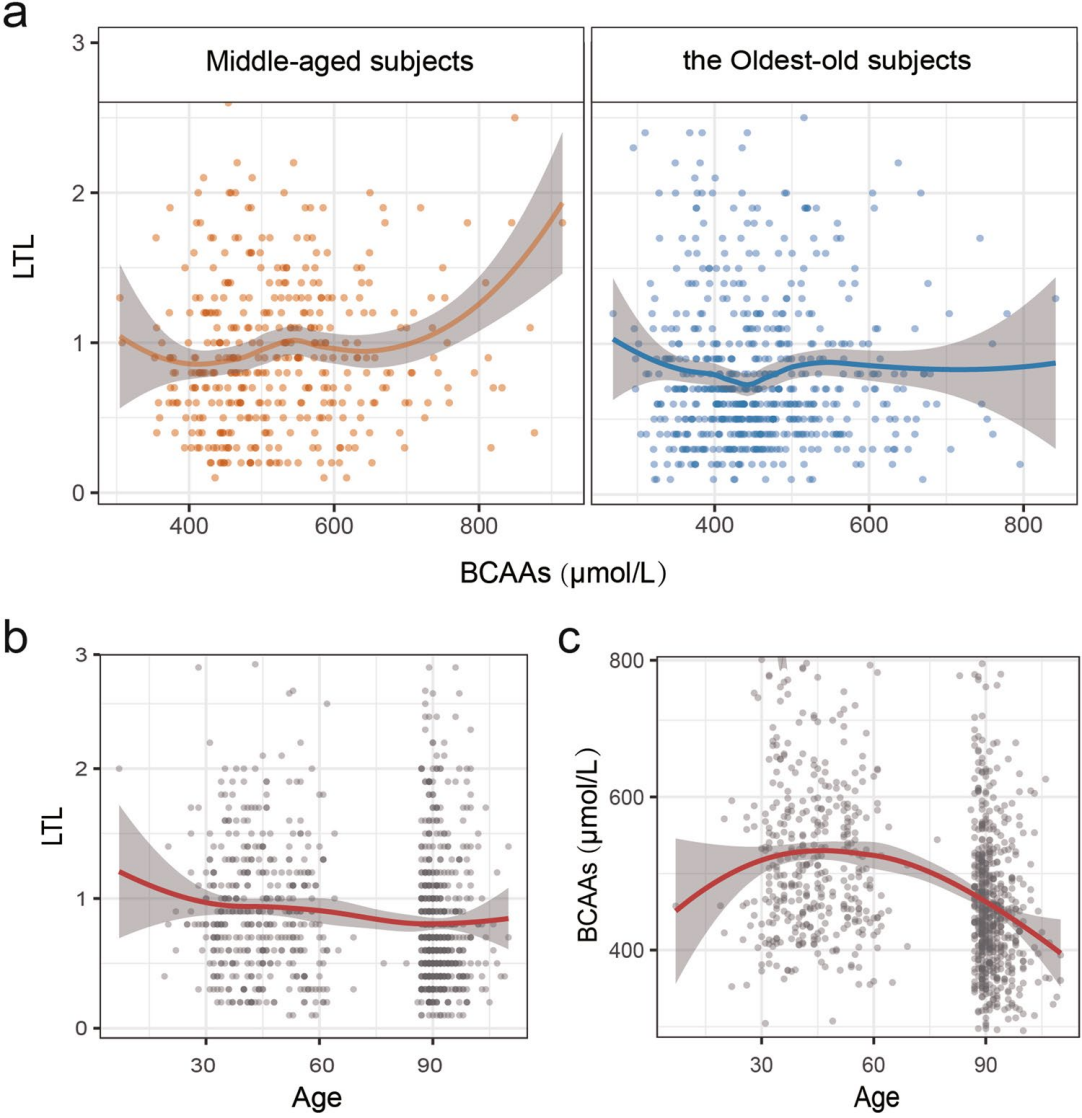
Associations of serum BCAAs, LTL and age. (**a**) Association between LTL and serum BCAAs. (**b**) Association between LTL and age. (**c**) Association between serum BCAAs and age. Smoothed lines between BCAAs, LTL and age were plotted with loess fitting using the R 3.4.3. The gray shaded area represents the 95% confidence interval. Each scatter represents a subject. BCAAs, branched-chain amino acids; LTL, leukocyte telomere length.

During adult aging, within-individual fasting serum BCAA levels may be fairly stable. Accordingly, the lower BCAA levels of the oldest old may be due to selective survival. In other words, higher mortality rates for middle-aged people with high BCAA levels may leave only the people who have always had relatively low BCAA levels to survive to 85+ and therefore be available for inclusion in this oldest old group. In our study, the positive association between BCAAs and LTL was not found in the oldest-old, which may due to a potential physiological shift from midlife (an anabolic state) to late life (a catabolic state) (Fig. 3). So, it is unreasonable that most of the medical guidelines for elderly subjects are still derived from studies of middle-aged. Likewise, there was no significant association between LTL and frailty score, which is consisted with other studies^19^, supporting the hypothesis that telomere length may be not a meaningful biomarker for frailty.

Although there are some limitations, such as a cross-sectional design and a limited sample size, this is the first study to explore the association between serum BCAAs, LTL and frailty based on the subjects from a longevity county in East Asia.

In the future, we will further control the confounding of malnutrition in the oldest old and exclude those subjects and test the reproducibility of our findings by a longitudinal study design with a larger sample size and multiple aging-related endpoints, including all-cause mortality.

## Conclusion

In summary, our findings suggest a potentially protective effect of circulating BCAAs on LTL and frailty based on the subjects from longevity county in East Asia and further indicate a potential synergetic interaction between BCAAs and LTL in healthy aging.

## Methods

### Study design and participants

A cross-sectional study was conducted in this study. The subjects were recruited by a quota and judgment sampling method from Yongfu County, Guangxi Province, which was qualified as the longevity town by Geriatric Society of China in 2007^20^. The oldest-old subjects were defined as successful agers aged above 85^21^. There are no lineal family members aged above 80 for 3 generations in middle-aged subjects.

### Information collection

The information of demographic data, lifestyle and health status was obtained using a standardized questionnaire. It was conducted by trained and qualified personnel. The peripheral fasting blood samples were separated for laboratory testing. Serum samples were isolated and stored at −80 °C until analysis.

### Determination of LTL

Genomic DNA was extracted by phenol-chloroform method. The DNA concentration and purity were assessed by measuring absorbance at 260 and 280 nm. The DNA integrity was tested by electrophoresis on a 1.0% agarose gel. The real-time qPCR method used for LTL measurement has been reported in detail previously^25^, which was widely used in large sample detection^26,27^. Briefly, LTL was measured in duplicate and expressed as a relative ratio of telomere repeat copy number to single copy gene (36B4) copy number compared to a reference standard (pooled genomic DNA generated within the lab). The primer sequences for the amplification reaction were 5′-GGTTTTTGAGGGTGAGGGTGAGGGTGAGGGTGAGGGT-3′ (F- Tel), 5′-TCCCGACTATCCCTATCCCTATCCCTATCCCTATCCCTA-3′ (R-Tel), 5′-CAGCAAGTGGGAAGGTGTAATCC-3′ (F-36B4), and 5′-CCCATTCTATCATCAACGGGTACAA-3′ (R-36B4).

### Determination of serum BCAAs concentration

A targeted metabolomics approach based on isotope dilution liquid chromatography tandem mass spectrometry (LC/MS/MS) method was used for the measurement of BCAAs concentration. The protocol has been reported detailly in our previous work^28,29^. Numerically, BCAAs means the sum of the concentrations of serum Val, Leu and Ile.

### Frailty score

To assess the health status in elderly people, a frailty score was defined as the proportion of accumulated deficits^30,31^ based on 24 general aging-related items (binary variables), including appearance features (11 items, e.g., dense wrinkles, yes or not), the ability of physical activity (3 items, e.g., walking on crutches, yes or not), the physical function (5 items, e.g., poor hearing, yes or not), the cognitive function (4 items, e.g., poor memory, yes or not) and disease (1 items, e.g., hypertension, yes or not) (Table S4). Each variable was coded as “0” (healthy) or “1” (unhealthy). The score can be calculated by counting the number of health deficits of each individual and dividing them by the total number of items measured^30^. The higher the score, the worse the physical condition. It is worth noting that this score assesses a more intuitive health status and reflects the characteristics of earlier aging compared with other studies.

### Statistical methods

The continuous variables of normal distribution were described as means ± SD and compared by student t test or covariance analysis; the data of non-normal distribution was expressed by median (quartile spacing) and compared by nonparametric test; the categorical variables were presented as frequencies (percentages) and compared by Pearson Chi-square test. The associations between BCAAs, LTL and frailty score were estimated by partial correlation and multiple linear regression. Further interaction analysis between BCAAs and LTL was estimated from the general linear model by converting BCAAs and LTL into binary variables with a median split method. Smoothed lines between BCAAs, LTL and age were plotted with loess fitting using the R 3.4.3. Heatmaps were plotted by R 3.4.3. All statistical tests were two-sided, and *P* < 0.05 was considered statistically significant. All analyses were performed using SPSS 22.0 (SPSS Inc.).

### Ethical statement

The study was approved by the Ethics Committee of Beijing Hospital and was conducted according to the guidelines in the Declaration of Helsinki. All participants or their guardians provided written informed consent.

## Supplementary Information

Supplementary Information.

## Data Availability

The datasets generated during and/or analysed during the current study are available from the corresponding author on reasonable request.
